# Mediastinal Epidermoid Cyst in a 5-Year-Old Girl

**DOI:** 10.1055/s-0037-1621707

**Published:** 2018-03-22

**Authors:** Keren A. Sloan, Kokila Lakhoo

**Affiliations:** 1 Department of Paediatric Surgery, John Radcliffe Hospital, Oxford, United Kingdom of Great Britain and Northern Ireland

**Keywords:** epidermoid cyst, pediatric, mediastinum

## Abstract

A 5-year-old girl was referred to our unit with an incidental finding of a lesion on the right hemithorax situated within the right atrial shadow. Computed tomography thorax showed a well-defined soft tissue lesion felt to be consistent with a bronchogenic cyst. The lesion was located in the posterior mediastinum, adherent to the diaphragm and inferior vena cava, but did not extend within the wall of the esophagus. It was entirely excised via video-assisted thoracoscopy converted to open thoracotomy. Histopathology confirmed an encapsulated nodular tissue measuring 2.5 × 2.5 × 2 cm lined by squamous type epithelium. Chronic inflammatory cells and foreign body giant cell reaction were found in the cyst wall. The appearances were that of a benign epidermoid cyst.

## Introduction

Mediastinal cysts are well-defined, round, epithelium-lined, fluid-containing lesions. 1 Of all mediastinal tumors, benign cystic lesions are reported to represent 12% to 20% of these. 1 2 The main differential diagnoses include a bronchogenic cyst, esophageal duplication, pleural, pericardial, and thymic cyst. 1 2 3 A cyst within the mediastinum is extremely rare and is not discussed as a differential in most published literature.

Histopathologic findings consistent with an epidermoid cyst include keratinized and stratified squamous epithelium, eosinophilic content within the wall, and *foreign body giant cells* can be seen. 4

## Case Report

**Fig. 1:**
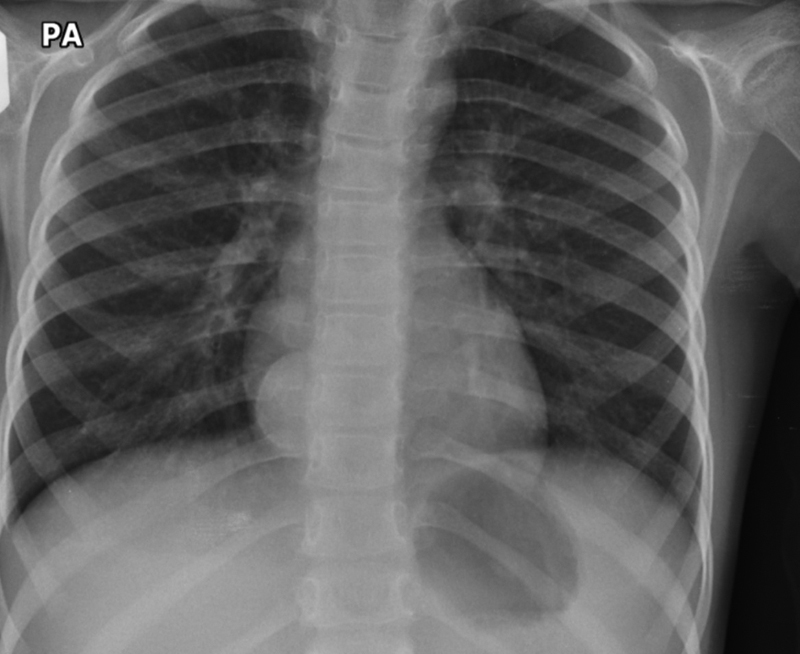
Chest X-ray showing a lesion in the right hemithorax situated within the right atrial shadow.

**Fig. 2:**
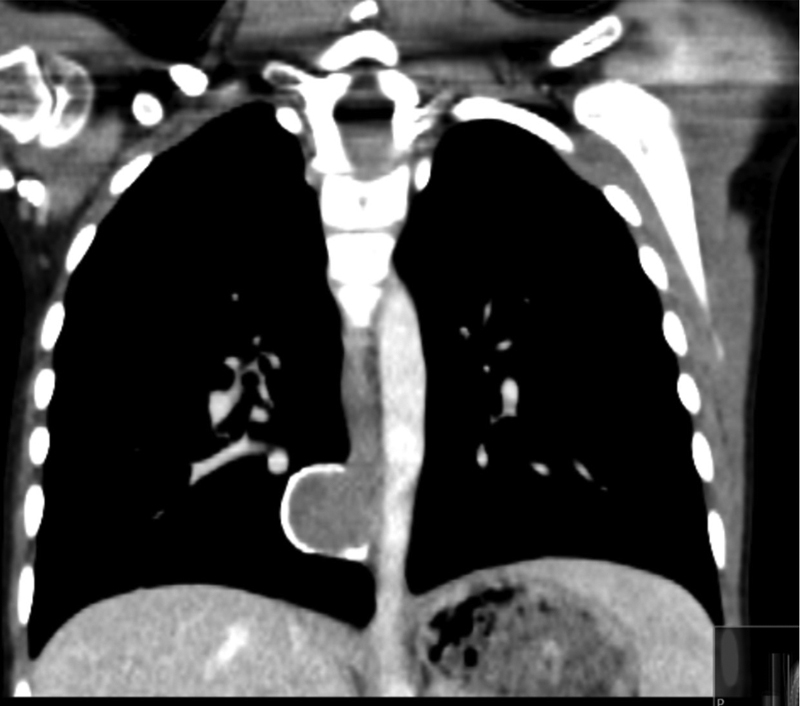
Computed tomography scan.

A 5-year-old girl was referred to our unit with an incidental finding of a lesion in the right hemithorax situated within the right atrial shadow ( Fig. 1 ). The mass was seen on a chest X-ray performed during an acute admission with bronchiolitis. There had been a normal chest X-ray performed 7 months previously. A computed tomography (CT) scan of the thorax confirmed the presence of a well-defined soft tissue lesion situated within the posterior mediastinum ( Fig. 2 ). The radiological appearance was felt to be consistent with a bronchogenic cyst.

There was no history of trauma or previous surgery; therefore, elective surgery was performed. After placement of a large nasogastric tube to delineate the esophagus, the patient was positioned on their left side with the right arm extended above the head. Surgery was initiated via a posterolateral video-assisted thoracoscopic surgery with a 30 degree scope and 5 mm instruments. Due to its adherence to the inferior vena cava and diaphragm, the case was converted to open thoracotomy. Thereafter, the mass which did not invade the wall of the esophagus was entirely excised. It contained sebum-like contents. The early postoperative course was unremarkable with full expansion of the lung on chest X-ray.

Histopathology confirmed an encapsulated nodular tissue measuring 2.5 × 2.5 × 2 cm and weighing 10 g. It was a large cystic space lined by squamous type epithelium, the cyst lumen comprised of mineralized debris. In the cyst wall, there were chronic inflammatory cells and foreign body giant cells. No atypia or malignancy was seen. The appearances were those of a benign epidermoid cyst.

The patient has made an excellent recovery and remains asymptomatic 2.75 years after the procedure.

## Discussion

Mediastinal epidermoid cysts in children are very rare with only four cases reported individually, 5 6 7 8 and another two within a larger case series. 9 Cases have been reported in various locations of the mediastinum including the thymus 10 and outwith the mediastinum like the gastrointestinal tract or spine. 11 12 All the patients with available demographics were adults. No case reports could be found within the pediatric population.

Of the mediastinal epidermoid cysts, case one was incidentally noted at routine pre-employment chest X-ray. At exploration, he was found to have a well-defined cyst attached to the right side of the pericardium. 5

Case two presented with hypotension and progressive dyspnea. On diagnostic workup, he was found to have a widened mediastinum, loculated pericardial effusion causing compression of the right atrium, and ventricle and a cystic lesion in the anterosuperior mediastinum. During surgery, a cystic mass attached to the pericardium was found. He later died secondary to respiratory complications. 6

Case three presented with chest pain. Chest X-ray and CT revealed a left basal opacity with a basal effusion. However, intraoperatively a large anterior mediastinal cyst attached to the pericardium was found. 7

The fourth case was reported in Russian and no further details could be obtained. 8

Like our case in all published cases, the histologic diagnosis of an epidermoid cyst was an incidental and unexpected finding.

The differential diagnosis includes bronchogenic, pleural, pericardial, and thymic cyst as well as esophageal duplication cyst. Histologically, an epidermoid cyst is lined with stratified squamous epithelium, contains a granular layer, lined with keratinous material.

This differs from a bronchogenic cyst, which is lined by ciliated or cuboidal epithelium and contains mucoid material. They can have tissues similar to those of normal bronchus, including hyaline cartilage, smooth muscle, elastic tissue, and mucus glands.

Esophageal duplication cysts are attached to the esophagus and have a double layer of smooth muscle and are lined with squamous or enteric epithelium. 1 2 3

Epidermoid cysts can be acquired following trauma or surgery secondary to implantation of epidermis into the dermis. However, the etiology of epidermoid cysts within the mediastinum remains uncertain. It is suspected to be congenital and develop from inclusion of ectodermal tissue remnants entrapped during development. 4 The cyst enlarges through proliferation of epidermal cells.

## Conclusion

Mediastinal epidermoid cysts in children may be asymptomatic or symptomatic secondary to local compression. The diagnosis usually cannot be expected on the basis of radiological imaging and is made by histopathology.
